# Mitochondrial Dysregulation Secondary to Endoplasmic Reticulum Stress in Autosomal Dominant Tubulointerstitial Kidney Disease – *UMOD* (ADTKD-*UMOD*)

**DOI:** 10.1038/srep42970

**Published:** 2017-02-21

**Authors:** Elisabeth Kemter, Thomas Fröhlich, Georg J. Arnold, Eckhard Wolf, Rüdiger Wanke

**Affiliations:** 1 Chair for Molecular Animal Breeding and Biotechnology, Gene Center, Ludwig-Maximilians-Universität München, D-81377 Munich, Germany; 2 Laboratory for Functional Genome Analysis (LAFUGA), Gene Center, Ludwig-Maximilians-Universität München, D-81377 Munich, Germany; 3 Institute of Veterinary Pathology, Center for Clinical Veterinary Medicine, Ludwig-Maximilians-Universität München, D-80539 Munich, Germany

## Abstract

‘Autosomal dominant tubulointerstitial kidney disease – *UMOD*’ (ADTKD-*UMOD*) is caused by impaired maturation and secretion of mutant uromodulin (UMOD) in thick ascending limb of Henle loop (TAL) cells, resulting in endoplasmic reticulum (ER) stress and unfolded protein response (UPR). To gain insight into pathophysiology, we analysed proteome profiles of TAL-enriched outer renal medulla samples from ADTKD-*UMOD* and control mice by quantitative LC-MS/MS. In total, 212 differentially abundant proteins were identified. Numerous ER proteins, including BiP (HSPA5), phosphorylated eIF2α (EIF2S1), ATF4, ATF6 and CHOP (DDIT3), were increased abundant, consistent with UPR. The abundance of hypoxia-inducible proteins with stress survival functions, i.e. HYOU1, TXNDC5 and ERO1L, was also increased. TAL cells in ADTKD-*UMOD* showed a decreased proportion of mitochondria and reduced abundance of multiple mitochondrial proteins, associated with disturbed post-translational processing and activation of the mitochondrial transcription factor NRF1. Impaired fission of organelles, as suggested by reduced abundance of FIS1, may be another reason for disturbed biogenesis of mitochondria and peroxisomes. Reduced amounts of numerous proteins of the OXPHOS and citrate cycle pathways, and activation of the LKB1-AMPK-pathway, a sensor pathway of cellular energy deficits, suggest impaired energy homeostasis. In conclusion, our study revealed secondary mitochondrial dysfunction in ADTKD-*UMOD.*

‘Autosomal dominant tubulointerstitial kidney disease – *UMOD*’ (ADTKD-*UMOD*; formerly uromodulin-associated kidney disease - UAKD) is a progressive dominant hereditary chronic kidney disease (CKD)^1^,^2^ caused by amino acid changing mutations of the gene encoding the glycoprotein uromodulin (UMOD). UMOD, which is expressed selectively in cells of the thick ascending limb of Henle’s loop (TAL) and of the early distal convoluted tubules^3^, is secreted and represents the most abundant protein in urine^1^. Impaired intracellular trafficking and intracellular accumulation of mutant UMOD protein causes TAL and consequently kidney dysfunction. Endoplasmic reticulum (ER) hyperplasia, induction of unfolded protein response (UPR) to increase folding capacity of the ER, and activation of NF-κB signalling in the TAL segment are the so far known pathomechanisms of ADTKD-*UMOD*^1^,^4^.

Disease progression leads to end-stage renal failure requiring renal replacement therapy between the ages of 25 and 70 years^5^. So far, only symptomatic therapeutic interventions can be done which do not prevent disease progression. A broader view into the molecular derangements in ADTKD-*UMOD* might be useful to develop novel therapeutic strategies. Therefore, we performed a holistic proteome analysis using quantitative liquid chromatography-tandem mass spectrometry (LC-MS/MS) of the affected kidney compartment in a well characterized mouse model for ADTKD-*UMOD*, the *Umod*^C93F^ mutant mouse line^6^. Results of the holistic proteome study were extended by Western blot analyses and immunohistochemical studies of *Umod*^C93F^ mutant mice and a second ADTKD-*UMOD* mouse model with a less pronounced disease phenotype, the *Umod*^A227T^ mutant mouse line.

## Results

### Quantitative LC-MS/MS analysis identified 212 differentially abundant proteins in ADTKD-*UMOD* kidneys

Label free quantification (LFQ) of proteins from the outer medulla kidney samples of four-month-old *Umod*^C93F^ mutant mice and wild-type controls was performed. In total, 10,727 peptides were identified (PEP value < 0.05) which could be assigned to 1,987 protein groups at a false discovery rate (FDR) <0.01. All identified proteins are listed in Supplementary Table 1.

Hierarchical clustering of LFQ values clearly separated the two genotype groups (Supplementary Fig. 1). In total, 212 proteins altered in abundance (adjusted p-value < 0.05; log2-fold change >|0.6|) were identified, 112 with increased and 100 with decreased abundance in *Umod*^C93F^ mutant vs. wild-type mice (Supplementary Table 2).

### Functional annotation clustering reveals alterations in the fractional composition of cellular organelles (ER, mitochondria, and peroxisomes) and in cellular energy metabolism

Differentially abundant proteins were clustered using DAVID analysis (Table 1, Supplementary Table 3) revealing that 30% of all identified differentially abundant proteins were located in mitochondria. Further cellular component clusters with differentially abundant proteins were ER and peroxisomes. Functionally annotated clusters of differentially abundant proteins in ADTKD-*UMOD* were related to aerobic respiration and citrate cycle, ATP biosynthesis and metabolism, catabolic process of glucose and other monosaccharides, oxidative phosphorylation (OXPHOS), and NADH/NADPH oxidoreductase activity. A further category of differentially abundant proteins contained extracellular matrix proteins.

### Disturbed ER homeostasis as a key feature of ADTKD-*UMOD*

**Figure 1:**
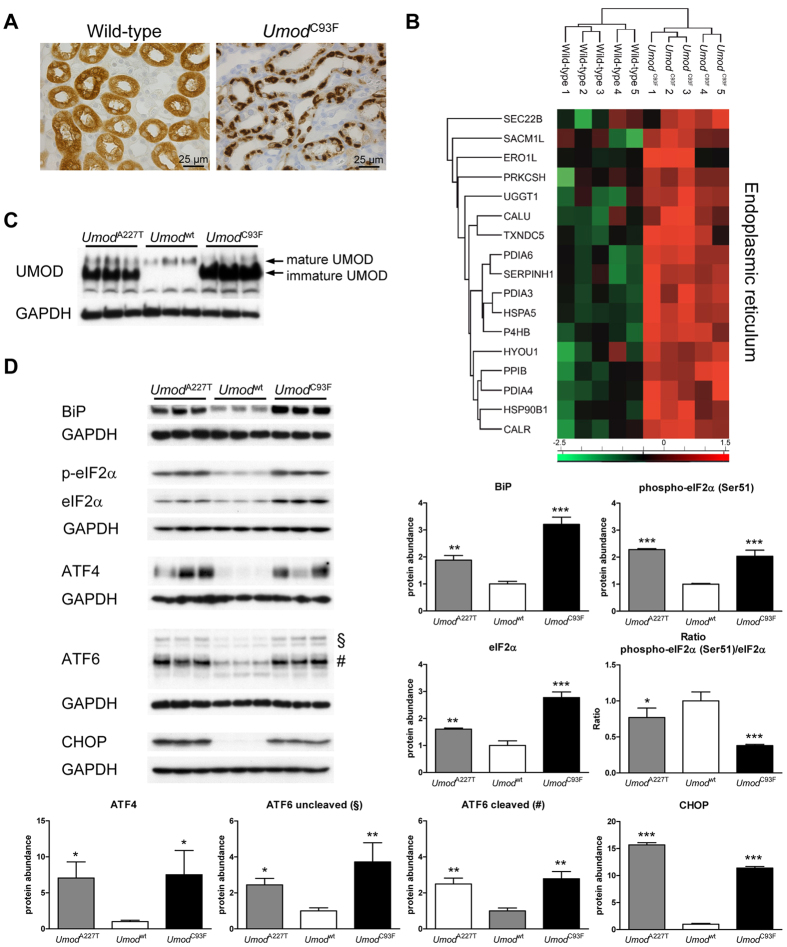
Disturbance of ER homeostasis in ADTKD-*UMOD*. (**A**) ADTKD-*UMOD* is characterized by maturation and trafficking defect of mutant UMOD and intracellular accumulation of UMOD in TAL cells. UMOD immunolocalization revealed a diffuse cytoplasmic staining with enforcement of the luminal membrane in TAL cells of a wild-type mouse. In contrast, TAL cells of an *Umod*^C93F^ mutant mouse displayed a strong paranuclear immunopositivity for UMOD. Wild-type: *Umod*^wt^ mouse; *Umod*^C93F^: homozygous *Umod*^C93F^ mutant mouse. Age of mice analysed: four months. Chromogen: DAB, nuclear staining: haemalum. (**B**) Heat map of relative expression values (z scores) showed differential abundance of several proteins localized in the ER. (**C**) In the outer medulla of *Umod* mutant mice of both mouse lines, a strong accumulation of immature UMOD was present. (**D**) Protein abundances of BiP, phospho-eIF2α, eIF2α, ATF4, both full-length (§) and cleaved activated (#) ATF6, and CHOP were increased in *Umod* mutant mice compared to wild-type mice. Signal intensities were corrected for GAPDH signal intensities of the same PVDF-membrane, which was stripped several times to facilitate the detection of multiple proteins. Mean of protein abundance of wild-type mice was set on a value of 1 [mean (wild-type) = 1]. Data are shown as means ± SD. One-way ANOVA with Newman-Keuls’s post hoc test: p vs. wild-type, *p < 0.05; **p < 0.01; ***p < 0.001.

Amino acid changing mutations in *UMOD* cause a defect of UMOD maturation and secretion, leading to intracellular perinuclear accumulation of immature UMOD (Fig. 1A) in the enlarged and stressed ER of TAL cells^1^,^6^. Significantly increased abundances of immature UMOD and of numerous ER-associated proteins were observed in the outer medulla of *Umod*^C93F^ mutant mice, with TXNDC5 showing the highest abundance increase (log2 fold change: 2.0) (Fig. 1B and C, Supplementary Table 4). Next, we evaluated ER stress pathways in more detail and revealed, besides an increase of BiP (HSPA5), an increase of the translation initiation factor eIF2α (EIF2S1) and its activated phosphorylated form (Fig. 1D) in *Umod* mutant mice. As shown by Western blotting, the amounts of eIF2α target proteins ATF4 and CHOP were also strongly increased. Furthermore, the full-length inactive and the cleaved active forms of ATF6 were increased abundant in ADTKD-*UMOD*. In summary, multiple proteins of the KEGG pathway ‘protein processing in ER’ were increased abundant in ADTKD-*UMOD*. These include proteins related to recognition by luminal chaperons (BiP, HYOU1, HSP90B1), to glycosylation (PDIA3, CALR, UGGT1), to intra-ER protein targeting (ERO1L, PDIA4, PDIA6, TXNDC5), and to the UPR pathway of ER stress (eIF2α, ATF4, ATF6, CHOP) (Supplementary Fig. 2).

### Reduced abundances of mitochondrial proteins and alterations in cellular localization indicate mitochondrial dysfunction in TAL cells of ADTKD-*UMOD* kidneys

**Figure 2:**
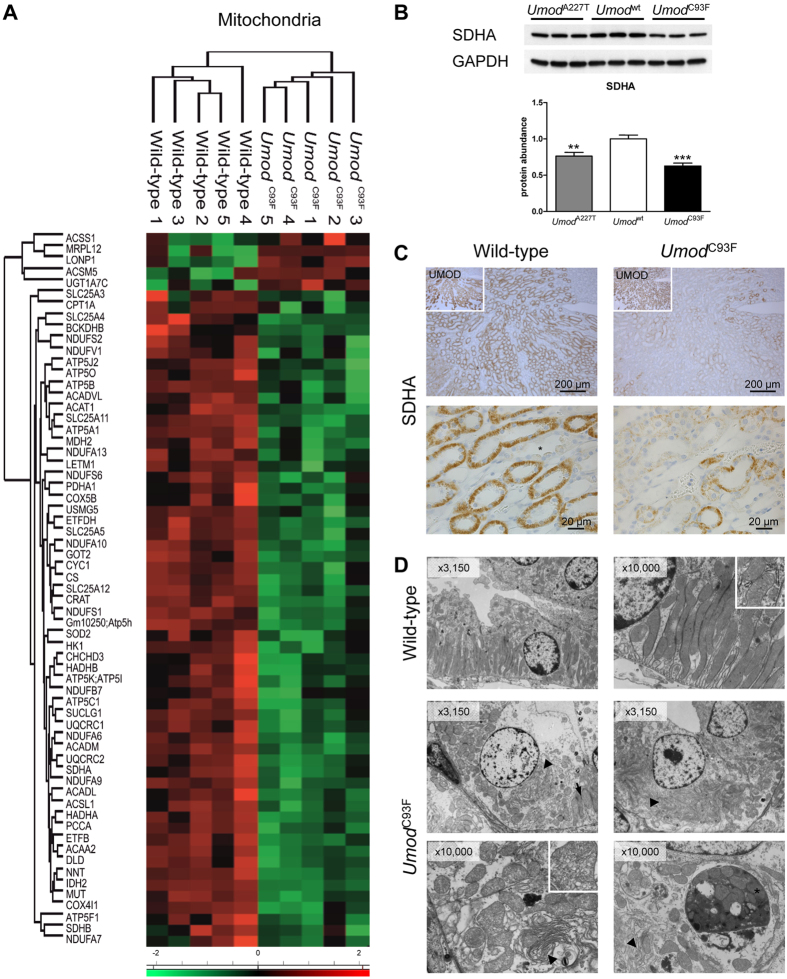
Disturbance of mitochondrial homeostasis in ADTKD-*UMOD*. (**A**) Z score heat map showed differentially abundant proteins localized in mitochondria. (**B**) In the outer medulla of *Umod* mutant mice, SDHA was less abundant compared to wild-type mice. Signal intensities were corrected for GAPDH signal intensities of the same PVDF-membrane. Mean of protein abundance of wild-type mice was set on a value of 1 [mean (wild-type) = 1]. One-way ANOVA with Newman-Keuls’s post hoc test: p vs. wild-type, **p < 0.01; ***p < 0.001. (**C**) Immunolocalization of SDHA revealed high abundance of SDHA in TAL segments of a wild-type mouse, whereas collecting duct cells (star) exhibited only weak SDHA immunostaining. Higher magnification of TAL cells demonstrated homogenous lamellar basal orientated cytoplasmic immunolocalization of SDHA in wild-type. In contrast, SDHA staining of TAL segments of the *Umod*^C93F^ mutant mouse was heterogeneous. Few TAL cells exhibited a SDHA staining intensity and pattern resembling that of wild-type TAL cells but were less compact, whereas the majority of TAL cells in the *Umod*^C93F^ mutant mouse had only sparse and more punctuated intracytoplasmic SDHA staining. Insert shows immunohistochemical localization of UMOD of the corresponding region of the outer medulla of the kidney. Wild-type: *Umod*^wt^ mouse; *Umod*^C93F^: homozygous *Umod*^C93F^ mutant mouse. Age of mice analysed: four months. Chromogen: DAB, nuclear staining: haemalum. (**D**) TAL cells of the wild-type mouse exhibited a well-formed basal labyrinth that stands perpendicularly on the basement membrane and where elongated mitochondria of crista type (insert) are parallel arranged. In TAL cells of *Umod*^C93F^ mutant mice, numerous predominantly paranuclear bundles (arrowhead) were present identified previously as hyperplastic ER^6^, which were heterogeneous pronounced in different TAL cells. In contrast to wild-type, TAL cells of the *Umod* mutant mouse had a poorly shaped (arrow) or even absent basal labyrinth. Further, mitochondria were loosely distributed in the cytoplasm, partly intervening in between the hyperplastic bundles of the perinuclear ER. Morphology of mitochondria varied in size and shape, and mitochondria were present exhibiting both crista and tubulus types (insert). Occasionally, mitophagy bodies were observed (star). Magnification is indicated in inserts. Age of mice analysed: four months.

Nearly one third of the differentially abundant proteins in ADTKD-*UMOD* are localized in mitochondria (Table 1), and most of them were reduced in abundance (Fig. 2A, Supplementary Table 5). The five mitochondrial proteins with increased abundance in ADTKD-*UMOD* were ACSS1, MRPL12, LONP1, ACSM5 and UGT1A7C.

The mitochondrial protein SDHA, which is part of OXPHOS respiratory complex II, was selected for immunolocalization. SDHA was significantly less abundant in the outer medulla of *Umod* mutant mice (Fig. 2B). In wild-type mice, SDHA was highly abundant in TAL segments (Fig. 2C, Supplementary Fig. 3A). Wild-type TAL cells showed a homogenous lamellar basal orientated cytoplasmic immunolocalization of SDHA, consistent with mitochondrial localization in TAL cells in the basal labyrinth as seen by ultrastructural analyses (Fig. 2D). In contrast, SDHA staining of TAL segments of *Umod* mutant mice was heterogeneous (Fig. 2C, Supplementary Fig. 3A). Few TAL cells of *Umod* mutant mice exhibited a SDHA staining intensity and pattern resembling that of wild-type TAL cells, whereas the majority of TAL cells in ADTKD-*UMOD* showed only sparse and more punctuated intracytoplasmic SDHA staining. Ultrastructural analyses of wild-type TAL cells revealed a distinctive basal labyrinth with numerous invaginations of the basal cell membrane, with exception of the perinuclear region where a small region of the cell membrane did not form invaginations (Fig. 2D). Close to the membrane invaginations, elongated mitochondria of the crista type were accumulated in parallel arrangement in the compartments of the basal labyrinth and looked homogeneous. In contrast, TAL cells of *Umod* mutant mice had a poorly shaped or even absent basal labyrinth. Furthermore, mitochondria were loosely distributed in the cytoplasm, partly intervening in between the hyperplastic bundles of the perinuclear endoplasmic reticulum. Morphology of mitochondria varied in size and shape, and mitochondria were present exhibiting both crista and tubulus types. Occasionally, mitophagy bodies were observed (Fig. 2D).

### Reduced abundance of FIS1 points to disturbed fission of mitochondria in ADTKD-*UMOD*

**Figure 3:**
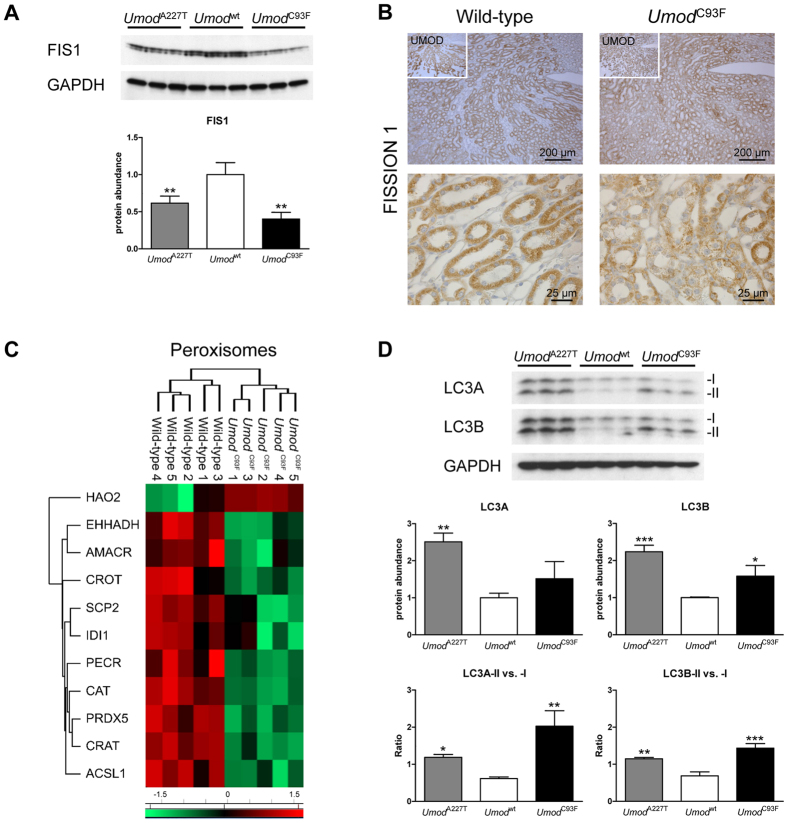
Reduction of FISSION 1 indicates disturbance in biogenesis and life cycle of mitochondria and peroxisomes, and increase of LC3A and LC3B indicates discreet increase of organelle autophagy. (**A**) In the outer medulla of *Umod* mutant mice, FIS1 was reduced abundant compared to wild-type mice, thereby the reduction was more pronounced in homozygous *Umod*^C93F^ than in homozygous *Umod*^A227T^ mutant mice. (**B**) In TAL cells of the wild-type mouse, FIS1 was highly abundant and were immunolocalized in the cytoplasm with nearly similar staining pattern as seen for SDHA, with high homogeneity between TAL segments. In contrast, FIS1 staining of TAL segments of the homozygous *Umod*^C93F^ mutant mouse was heterogeneous, where only some TAL cells had a similar FIS1 staining pattern as seen in TAL cells of the wild-type mouse. In numerous TAL cells of the homozygous *Umod*^C93F^ mutant mouse, FIS1 seemed to be displaced to the cytoplasmic borders of the cells with nearly negative staining predominantly of the paranuclear regions of TAL cells. Insert shows immunohistochemical localization of UMOD of the corresponding region of the outer medulla of the kidney. Wild-type: *Umod*^wt^ mouse; *Umod*^C93F^: homozygous *Umod*^C93F^ mutant mouse. Age of mice analysed: four months. Chromogen: DAB, nuclear staining: haemalum. (**C**) Z score heat map showed differentially abundant proteins localized in peroxisomes. (**D**) In the outer medulla of *Umod* mutant mice, LC3A and LC3B were increased abundant compared to wild-type mice. Further, LC3-II vs. -I ratios of LC3A and LC3B were significantly increased in *Umod* mutant mice indicating an increase of autophagy due to increased conversion of isoform I (16 kDa) into isoform II (14 kDa) of LC3. (**A & D**) Signal intensities of immunoblots were corrected for GAPDH signal intensities of the same PVDF-membrane. Mean of protein abundance of wild-type mice was set on a value of 1 [mean (wild-type) = 1]. Data are shown as means ± SD. One-way ANOVA with Newman-Keuls’s post hoc test: p vs. wild-type, *p < 0.05; **p < 0.01; ***p < 0.001.

The life cycle of mitochondria involves biogenesis from newly synthesized proteins, fusion and fission to regulate mitochondrial dynamics, and mitophagy as final step of quality control^7^,^8^. In addition, fission provides a mechanism for selective clearance of damaged components in mitochondria thereby extending their life span. Since FIS1 is essentially involved in mitochondrial fission, abundance and localization of this protein was analysed. FIS1 was significantly reduced in the outer medulla of ADTKD-*UMOD* compared to wild-type kidneys (Fig. 3A). In TAL cells of wild-type mice, FIS1 was highly abundant and localized in the cytoplasm with a similar staining pattern as seen for SDHA and little variability among TAL cells (Fig. 3B, Supplementary Fig. 3B). In contrast, FIS1 staining of TAL segments of *Umod* mutant mice was heterogeneous, with only some TAL cells showing a similar FIS1 staining pattern as seen in wild-type mice. In numerous ADTKD-*UMOD* TAL cells, FIS1 staining was limited to the cytoplasmic borders with nearly negative cytoplasmic staining of the perinuclear region. Impaired fission is also supported by the altered morphology of mitochondria in ADTKD-*UMOD* TAL cells (Fig. 2D).

Fission is also an essential step in the biogenesis and life cycle of peroxisomes^9^,^10^. Peroxisomes are dynamic organelles involved in lipid metabolism which respond to environmental stimuli. Interestingly, 10 peroxisomal proteins were less abundant in the outer medulla of ADTKD-*UMOD* compared to control kidneys, and only one (HAO2) was more abundant (Fig. 3C, Supplementary Table 6), suggesting impaired biogenesis of peroxisomes.

### Autophagy of mitochondria as a putative adverse outcome of impaired life cycle mitochondrial dynamic in ADTKD-*UMOD*

To address changes in mitophagy, LC3A and LC3B as markers for organelle autophagy were analysed (Fig. 3D). Kidneys of *Umod* mutant mice exhibited an increased abundance of LC3A and LC3B. Both proteins exist in isoforms I and II. During autophagosome formation and maturation, isoform I (16 kDa) is converted to isoform II (14 kDa). In *Umod* mutant mice, the ratio of isoform II/isoform I was significantly increased for both LC3A and LC3B, suggesting increased organelle autophagy in ADTKD-*UMOD*. Of note, occasionally, mitophagy bodies were observed in TAL cells of *Umod* mutant mice (Fig. 2D).

### Impaired maturation and processing of NRF1 suggests disturbed transcriptional regulation of mitochondrial biogenesis

**Figure 4:**
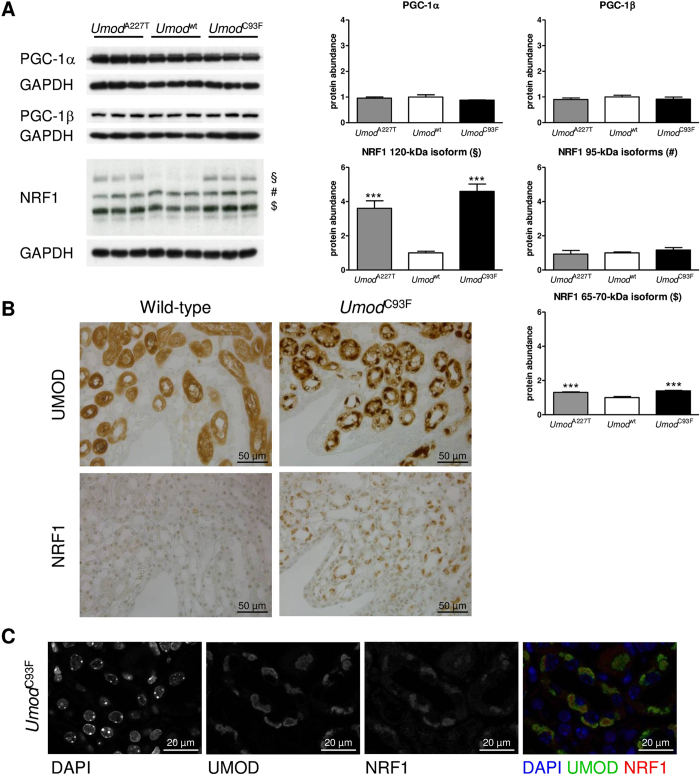
Disturbed post-translational processing of NRF1 abrogates transcriptional regulation of mitochondrial biogenesis by NRF1. (**A**) Relative protein abundances of PGC-1α, PGC-1β and NRF1 in the outer medulla of *Umod* mutant mice and wild-type mice. § 120-kDa inactive ER-resident NRF1 glycoprotein, # 95-kDa full-length non-glycosylated protein (precursor) and/or active de-glycosylated isoforms of NRF1, $ 65–70-kDa isoform of NRF1. Signal intensities were corrected for GAPDH signal intensities of the same PVDF-membrane. Mean of protein abundance of wild-type mice was set on a value of 1 [mean (wild-type) = 1]. Data are shown as means ± SD. One-way ANOVA with Newman-Keuls’s post hoc test: p vs. wild-type, *p < 0.05; **p < 0.01; ***p < 0.001. Wild-type: *Umod*^wt^ mice; *Umod*^C93F^: homozygous *Umod*^C93F^ mutant mice; *Umod*^A227T^: homozygous *Umod*^A227T^ mutant mice. (**B**) Immunohistochemical localization of NRF1. In TAL cells of the wild-type mouse, NRF1 was discreet abundant in nuclei. In contrast, the *Umod*^C93F^ mutant mouse exhibited additionally to the discreet nuclear staining a distinct paranuclear immunopositivity of variable intensities of NRF1 in TAL cells. Cells of TAL segment were identified by UMOD immunostaining. Wild-type: *Umod*^wt^ mouse; *Umod*^C93F^: homozygous *Umod*^C93F^ mutant mouse. Age of mice analysed: four months. Chromogen: DAB, nuclear staining: haemalum. (**C**) Co-localization of uromodulin and NRF1 in TAL cells of a four-month-old homozygous *Umod*^C93F^ mutant mouse, studied by multicolour immunofluorescence analysis. UMOD is known to be accumulated in the hyperplastic endoplasmic reticulum (ER) of TAL cells in ADTKD-*UMOD*. Co-localization of NRF1 with UMOD is indicative for accumulation of NRF1 also in the ER of TAL cells. Some nuclei exhibited a discreet NRF1 immunofluorescence with distinct nuclear foci. DAPI: nuclear marker.

Mitochondrial biogenesis requires transcription of genes encoding mitochondrial proteins. In this context, PGC-1α and PGC-1β play a crucial role as transcriptional co-activators^11^. The abundances of PGC-1α and PGC-1β in kidney samples were not different between *Umod* mutant and wild-type mice (Fig. 4A). Next, we analysed NRF transcription factors, which are co-activated by PGC-1α^11^. The glycoprotein NRF1 can occur in various kDa sizes due to complex post-translational processing and modification (glycosylation/deglycosylation, proteolytic cleavage) which are relevant for its transactivation activity^12^. After translation in a 95-kDa full-length protein, NRF1 becomes glycosylated to a non-active 120-kDa isoform in the ER lumen. Thereafter, NRF1 is translocated from the ER to the nucleus as a de-glycosylated active 95-kDa transcription factor. Further, NRF1 can be subjected to proteolytic processing resulting in shorter isoforms. In ADTKD-*UMOD* affected kidneys, the abundance of the glycosylated 120-kDa NRF1 isoform and – to a lesser extent – of a 65–70-kDa NRF1 isoform was increased, whereas the abundance of the 95-kDa full-length non-/de-glycosylated NRF1 isoform was similar between *Umod* mutant mice and wild-type mice (Fig. 4A). Immunolocalisation revealed a discreet nuclear staining of NRF1 in TAL cells of wild-type mice (Fig. 4B). In contrast, *Umod* mutant mice exhibited additionally to the discreet nuclear staining a distinct paranuclear immunopositivity of variable intensities of NRF1 in TAL cells. As shown by multicolour immunofluorescence analysis, paranuclear accumulation of NRF1 was co-localized with UMOD (Fig. 4C), the latter was previously demonstrated to be accumulated in the enlarged ER of TAL cells in ADKTD-*UMOD*^6^. Entrapment of NRF1 in the hyperplastic ER of TAL cells in ADTKD-*UMOD* associated with accumulation of glycosylated 120-kDa NRF1 isoform is consistent with impaired maturation, processing and activation of NRF1 in ADTKD-*UMOD*.

### Disturbed cellular energy homeostasis in ADTKD-*UMOD* affected kidneys

**Figure 5:**
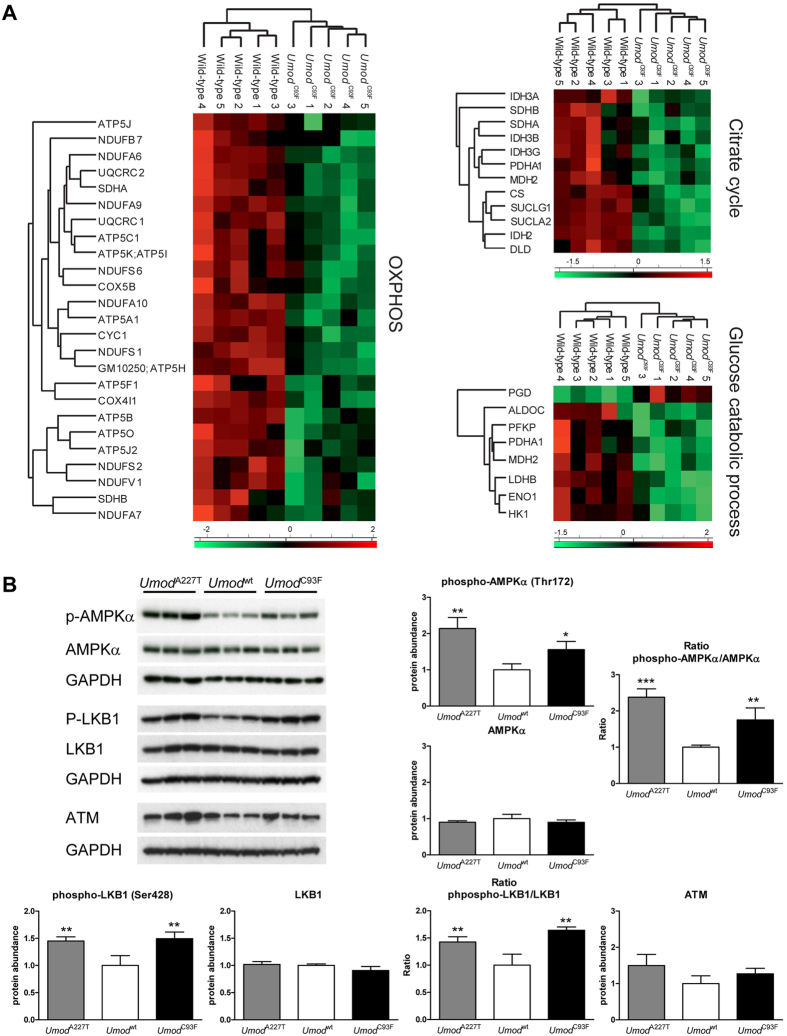
Disturbed cellular energy homeostasis in ADTKD-*UMOD* affected kidneys. (**A**) Z score heat maps showed differentially abundant proteins involved in OXPHOS, citrate cycle and glucose catabolic process. (**B**) The phosphorylation of AMPKα was increased in the outer medulla of *Umod*^A227T^ and *Umod*^C93F^ mutant mice compared to wild-type mice, indicating an impaired cellular energy homeostasis in ADTKD-*UMOD*. Protein abundance of AMPKα was similar between different genotypes. The phosphorylation of LKB1 was increased in the outer medulla of *Umod*^A227T^ and *Umod*^C93F^ mutant mice compared to wild-type mice, further supporting existence of energy depletion in ADTKD-*UMOD*. Protein abundance of LKB1 was similar between different genotypes. Also ATM abundance was similar irrespective of genotype. Signal intensities were corrected for GAPDH signal intensities of the same PVDF-membrane. Mean of protein abundance of wild-type mice was set on a value of 1 [mean (wild-type) = 1]. Data are shown as means ± SD. One-way ANOVA with Newman-Keuls’s post hoc test: p vs. wild-type, *p < 0.05; **p < 0.01; ***p < 0.001.

As powerhouse of the cell, energy in form of ATP is mainly derived by aerobic respiration in mitochondria^10^. Numerous proteins of both OXPHOS (25 differentially abundant proteins) and citrate cycle (12 proteins) were strongly reduced in abundance in ADTKD-*UMOD*, affecting all five mitochondrial respiratory complexes of OXPHOS (Fig. 5A, Supplementary Table 7). Eight detected differentially abundant proteins were proteins involved in glucose metabolic process, thereof PGD showed increased and the other seven reduced abundance in ADTKD-*UMOD* (Fig. 5A, Supplementary Table 7). Three of them (HK1, MDH2 and PDHA1) are localized in mitochondria. PGD has a role in oxidative pentose phosphate pathway. Of note, GAPDH was similar abundant in LC-MS/MS analyses of outer medulla of wild-type and *Umod* mutant mice. Decreased abundances of numerous proteins involved in glycolysis, OXPHOS and citrate cycle indicate that several metabolic pathways for cellular energy supply were negatively affected. Therefore, we next analysed the cellular energy sensor AMP-activated protein kinase (AMPK), which is activated by phosphorylation in response to energy depletion^13^. Consistent with the assumption of a negative energy balance in ADTKD-*UMOD* affected kidneys, the levels of phosphorylated AMPKα were significantly increased (Fig. 5B). Next, we analysed the cellular energy sensing signalling kinase LKB1 which is able to phosphorylate and thereby to activate AMPK^14^. The levels of phosphorylated LKB1 were distinctly higher in kidneys of *Umod* mutant mice than in controls, supporting activation of the LKB-AMPK pathway in response to disturbed energy homeostasis in ADTKD-*UMOD* affected kidneys (Fig. 5B). LKB1 or AMPK can be activated by ATM, a critical early DNA damage response protein^14^. However, the abundance of ATM was similar in kidneys of *Umod* mutant and wild-type mice.

### Early signs of renal fibrotic processes in ADTKD-*UMOD*

Ten extracellular matrix proteins with increased abundances were identified in the outer medulla of ADTKD-*UMOD* affected kidneys compared to wild-type controls (Supplementary Fig. 4, Supplementary Table 8). Highest increase in the abundance exhibited COL1A1, COL14A1 and LUM (log2 fold change: 2.5, 2.1 and 1.8, respectively).

## Discussion

ADTKD-*UMOD* is caused by amino acid changing mutations of UMOD which lead to a pronounced protein maturation and secretion defect of both mutated and native UMOD, massive accumulation of immature UMOD in the enlarged ER of TAL cells and strongly reduced urinary UMOD excretion^1^,^6^. Protein maturation defects of UMOD cause a strong disturbance of the ER homeostasis, ER stress and induction of UPR. These events are associated with pronounced morphological alterations in TAL cells like massive enlargement of the ER.

**Figure 6:**
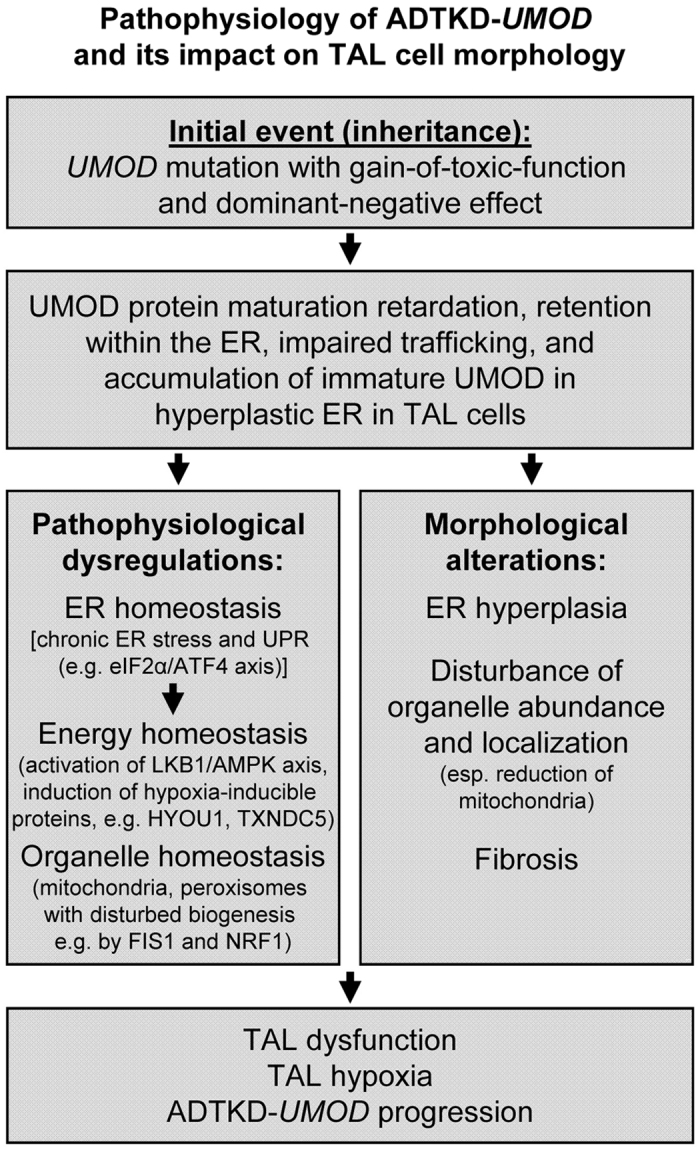
Flowchart of the pathophysiology of ADTKD-*UMOD* and its impact on TAL cell morphology.

Based on the results of our holistic proteome analysis of the ADTKD-*UMOD* mouse model, we can extend the flowchart of pathophysiological events and their impact on TAL cell morphology and function in ADTKD-*UMOD* (Fig. 6).

Upregulation of numerous ER proteins, involved in protein folding, quality control, intra-ER-transport and glycosylation, may be interpreted as an attempt of the TAL cells to promote maturation and urinary secretion of mutant UMOD protein. These cellular efforts are at least partially successful, as demonstrated by low amounts of UMOD in urine of homozygous *Umod* mutant mice^6^. However, ER overload also negatively affects maturation and processing of native UMOD protein^1^,^6^,^15^ and of other proteins like the mitochondrial transcription factor NRF1, thereby inhibiting its activation and nuclear translocation (see below).

Long-lasting ER stress and UPR resulted in strong activation of the NF-κB pathway^4^ and, as shown in the present study, of the eIF2α-ATF-CHOP pathway and – to a lesser extent – of the ATF6 pathway. Increased levels of ER proteins ERO1L, HYOU1 and TXNDC5, which are induced as cellular response to hypoxia^16^,^17^,^18^, might have protective effects on TAL cells. Stress survival functions under hypoxia were described for HYOU1 and TXNDC5 18,19. Of note, CHOP induces upregulation of the anti-apoptotic factor HYOU1^20^, and HYOU1 can control the activity of the pro-apoptotic protein CHOP^21^. Up-regulation of survival factors like HYOU1 or TXNDC5 might thus protect TAL cells from apoptosis despite increased CHOP abundance. This might contribute to the slow progression of ADTKD-*UMOD* in our *Umod* mutant mice and in affected patients.

How can ER hyperplasia and chronic ER stress affect mitochondria? Biogenesis of mitochondria requires transcriptional activation of genes encoding mitochondrial proteins^7^. The transcription factor PGC-1 and its essential co-activator NRF1 play a critical role in mitochondrial biogenesis^22^. Accumulation of the inactive glycosylated isoform of NRF1 in the hyperplastic ER of TAL cells likely contributes to reduced mitochondrial mass and dysfunction in ADTKD-*UMOD*. In addition, spatial interaction with the ER is relevant for mitochondrial biogenesis by fission^10^,^23^. Mitochondria can dynamically respond to environmental stimuli by fusion and fission^9^,^10^. These processes are also important for mitochondria to get rid of damaged mitochondrial proteins and thereby to prolong life time of mitochondria^8^. FIS1 in TAL cells of *Umod* mutant mice was decreased in abundance and showed altered cellular distribution, pointing to reduced fission activity of mitochondria. This assumption is supported by the heterogeneous morphology of mitochondria in TAL cells of *Umod* mutant mice. The abundance of LONP1, a mitochondrial protease involved in degradation of damaged proteins and in mitochondrial quality control under stress conditions^24^,^25^, was increased in ADTKD-*UMOD* suggesting the occurrence of stressed mitochondria, which might be more susceptible to enter the mitophagy process.

Besides its impact on mitochondrial biogenesis, ER stress can also influence the function of mitochondria. The physical contact site between ER and mitochondria is the mitochondria-associated membrane (MAM), a critical signalling platform facilitating calcium and lipid transfer between these organelles^26^. The MAM alters its set of regulatory proteins and functions during cellular stress. Several MAM proteins were differentially abundant in ADTKD-*UMOD*. The abundance of ACAT1, often used as marker of MAM (reviewed in ref. 26), was decreased, while the MAM proteins ERO1L^27^ and BiP^26^ were increased abundant in ADTKD-*UMOD*. Altered levels of several MAM proteins suggest disturbed signalling between chronically stressed ER and mitochondria.

Strong reduction of proteins of OXPHOS and citrate cycle, and an increase of numerous hypoxia-inducible proteins in ADTKD-*UMOD* point to impaired energy homeostasis of TAL cells. This is supported by increased activation of the LKB1-AMPK axis, the sensor pathway of cellular energy status^14^. Interestingly, PGD, a central enzyme of the pentose phosphate pathway, was more abundant in *Umod*^C93F^ mutant mice than in wild-type controls. Activation of the pentose phosphate pathway could provide an alternative energy supply to mitochondrial ATP synthesis (Warburg effect)^28^. LONP1, which was higher abundant in ADTKD-*UMOD*, can favour the switch from a respiratory to a glycolytic metabolism^25^,^29^.

Besides their function as powerhouse of the cell, mitochondria are critically involved in intracellular calcium handling and apoptosis signalling^30^,^31^. Mitochondrial dysfunction is known to play a critical role in the pathogenesis of kidney diseases^30^. Ultrastructural studies of TAL cells of ADTKD-*UMOD* patients described TAL cells with fibrillar material mostly evident in ER with smaller area overrunning mitochondria^32^ or TAL cells containing hyperplastic ER bundles with few intervening mitochondria alternating with areas of dilated ER containing storage material^33^. These reports underline the clinical relevance of mitochondrial alterations uncovered by the present study in ADTKD-*UMOD* mouse model. Mitochondrial changes are already present in an early stage of the disease and likely contribute to TAL dysfunction and disease progression.

In conclusion, the proteomic alterations identified in this study mirror the pronounced structural alterations of TAL cells in the ADTKD-*UMOD* mouse model, which are also described in human patients. Apart from well-known ER stress, mitochondrial dysfunction and altered energy homeostasis are newly identified important components in ADTKD-*UMOD* pathophysiology and may provide novel targets for so far not available therapeutic strategies.

## Materials and Methods

### Mouse models for ADTKD-*UMOD*

All animal experiments were approved by local government authorities (Regierung von Oberbayern) in accordance with the German Animal Welfare Act and conformed to the *Guide for the Care and Use of Laboratory Animals* as published by the US National Institutes of Health. The two well established mouse models of ADTKD-*UMOD, Umod*^A227T^ and *Umod*^C93F^, were used^6^,^15^. Both *Umod* mutant mouse lines exhibit key features of ADTKD-*UMOD* like dominant-negative effect of mutant UMOD protein with maturation defect and retention of UMOD in the hyperplastic endoplasmic reticulum of TAL cells and impaired kidney function with mild defect in urinary concentration ability, reduced fractional excretion of uric acid, and reduced uromodulin excretion. Although *Umod*^A227T^ and *Umod*^C93F^ mutant mice exhibit similar disease phenotype, *Umod*^C93F^ mutant mice are earlier and more severely affected by a more pronounced mutant UMOD maturation defect and TAL dysfunction. Homozygous mutant mice exhibit a more pronounced disease phenotype than heterozygous mutants of the same line. A correlate of the *Umod*^C93F^ mutation exists in humans, where ADTKD-*UMOD* affected patients were identified harbouring disease-causing mutations p.C94W (human *UMOD*^C94^ corresponds to murine *Umod*^C93^) and p.C106G, p.C106Y, p.C106F (human *UMOD*^C106^ corresponds to murine *Umod*^C105^) leading to disruption of a putative disulphide bond C94-C106 in human UMOD (respectively C93-C105 in murine UMOD) (Wake Forest Inherited Kidney Disease Registry, http://www.ukdcure.org/mutation_catalog)^6^. Both *Umod*^A227T^ and *Umod*^C93F^ mutant mouse lines were maintained in the C3HeB/FeJ (C3H) genetic background and housed in a specific pathogen-free mouse facility. All mice had access to food (V1124-3, Ssniff, Soest, Germany) and water ad libitum and mouse husbandry was performed under standard environment conditions (22 ± 2 °C, 40–50% relative humidity, 12 h light/dark cycle).

### Sample preparation for proteome analyses

TAL segments are the primary site of injury in ADTKD-*UMOD* and the TAL segment density in the kidney is highest in the outer medullar region. Using the outer medulla was proven to enable evaluation of pathophysiology of ADTKD-*UMOD* in a previous study^4^. *In vitro* models using mutant UMOD transfected cells were previously described by others to do not mirror the key cellular hallmarks of ADTKD-*UMOD*, which are mutant UMOD aggregation, ER membrane expansion, and ER dilation, and was therefore reported to be not suitable to analyse potential disease mechanisms downstream of mutant UMOD retention in ADTKD-*UMOD*^1^. The outer medulla was prepared as described^4^. Briefly, each fresh kidney of four-month-old mice was cut along the midsagittal plane into two halves and further into transverse slices. The outer medulla were then received by removing the cortex and inner medulla, shock frozen on dry ice and stored at −80 °C until further use for mass spectrometry or Western Blot analyses. Always the whole preparation of the outer medulla of one kidney was used for sample homogenization. Four-month-old mice were used in this study. At this age the ADTKD-*UMOD* phenotype is already clinically present in *Umod* mutant mice of both lines, but is in an early disease stage where no progressed morphological kidney alterations like interstitial fibrosis, tubular atrophy, or infiltrates of inflammatory cells are present (Supplementary Fig. 5).

### Sample preparation for and performance of quantitative LC-MS/MS

The outer medulla of five four-month-old male homozygous *Umod*^C93F^ mutant mice and age-matched wild-type littermate controls was homogenized in a MS lysis buffer containing 8 M urea and 0.4 M NH_3_HCO_3_. Protein concentrations were determined with a 660 nm assay (Thermo-Scientific). Prior to tryptic digestion, protein concentrations were adjusted to 1 mg/ml with MS lysis buffer. DTE to a final concentration of 4 mM was added to 100 μg protein in 100 μl (conc. 1 mg/ml), and incubated for 30 min at room temperature. Next, cysteine residues were blocked by addition of iodoacetamide to a final concentration of 8 mM and further incubated for 30 min in the dark. After dilution with water to give a concentration of 1 M urea, 2 μg porcine trypsin (Promega, Madison, WI, USA) was added and incubated overnight at 37 °C.

Peptide samples were analysed by LC-MS/MS analysis using an UltiMate 3000 nano LC system (Thermo Scientific, Waltham, MA, USA) connected to a TripleTOF 5600+ mass spectrometer (Sciex, Concord, Canada). 2.5 μg of peptides diluted in chromatography solvent A (0.1% formic acid) were transferred at a flow rate of 30 μl/min to a trap column (Acclaim PepMap 100, μ-Precolumns, 5 mm × 300 μm, 5 μm particles, Thermo Scientific) and separated at a flow rate of 200 nL/min with a 50 cm separation column (Acclaim PepMap RSLC C18, 75 μm × 50 cm, 2 μm, Thermo Scientific). Consecutive gradients, from 2% to 25% solvent B (0.1% formic acid, 100% ACN) in 260 min and from 25% to 50% solvent B in 60 min were used. MS spectra were acquired (positive ion mode) in cycles of one MS scan (mass range m/z 200–1800) followed by ten data dependent CID MS/MS scans (collision energy 45).

### Data analysis of quantitative LC-MS/MS derived holistic proteome analysis

MS wiff files were processed using MaxQuant version 1.5.1. As protein databases, the Mus musculus subset of the UniProt and the MaxQuants common contaminants database was used. For database searches the MaxQuant default values for Sciex TOF instruments and a protein FDR of 1% was applied. The quantification was performed with the label-free quantification (LFQ) strategy implemented in MaxQuant. Statistics as well as the heatmap generation was performed using the Perseus module, part of the MaxQuant software pipeline. In cases where proteins were detected in at least three samples of one group, the “imputation form normal distribution” feature implemented in Perseus was used. For statistical evaluation a two sided T-test and a permutation-based FDR estimation was performed.

To identify functional relationships among differentially abundant proteins identified by quantitative LC-MS/MS analysis, we used the DAVID bioinformatics online tool for functional annotation clustering^34^. The annotation categories used in our DAVID cluster enrichment analysis were the functional category SP_PIR_KEYWORDS, UP_SEQ_FEATURE, the gene ontology terms GOTERM_BP_FAT, GOTERM_CC_FAT, and GOTERM_MF_FAT, and KEGG_PATHWAY; analysis was performed with high classification stringency. We considered functional clusters having enrichment score of greater than 2 and individual Fisher’s exact and of Benjamini *P* values of less than 0.05 for each term in the cluster.

### Western blot analyses

The outer medulla of four-month-old homozygous *Umod*^C93F^ mutant mice, homozygous *Umod*^A227T^ mutant mice and wild-type mice was homogenised in Laemmli extraction buffer and protein concentration was determined by BCA assay. 15 μg of denatured tissue lysate per lane was separated on SDS-polyacrylamide minigels and blotted on PVDF membranes. Equal loading was controlled by Ponceau staining. The following primary antibodies were used: rabbit monoclonal antibody against phospho-AMPKα (Thr172) (40H9, no. 2535, Cell Signaling), rabbit polyclonal antibody against AMPKα (no. 2532, Cell Signaling), rabbit monoclonal antibody against ATF4 (D4B8, no. 11815, Cell Signaling), mouse monoclonal antibody against ATF6 (70B1413.1, no. NBP1-40256, novusbio), rabbit monoclonal antibody against ATM (D2E2, no. 2873, Cell Signaling), rabbit polyclonal antibody against BiP (no. 3183, Cell Signaling), rabbit monoclonal antibody against CHOP (D46F1, no. 5554, Cell Signaling), rabbit polyclonal antibody against FIS1 (no. GTX111010, GeneTex), rabbit monoclonal antibody against GAPDH (D16H11, no. 5174, Cell Signaling), rabbit monoclonal antibody against LC3A (D50G8, no. 4599, Cell Signaling), rabbit monoclonal antibody against LC3B (D11, no. 3868, Cell Signaling), rabbit monoclonal antibody against phospho-LKB1 (Ser428) (C67A3, no. 3482, Cell Signaling), rabbit monoclonal antibody against LKB1 (D60C5, no. 3047, Cell Signaling), rabbit polyclonal antibody against NRF1 (no. 12381, Cell Signaling), rabbit polyclonal antibody against PGC-1α (no. NBP1-04676, novusbio), mouse monoclonal antibody against PGC-1β (no. sc-373771, Santa Cruz), rabbit monoclonal antibody against SDHA (D6J9M, no. 11998, Cell Signaling), and rabbit polyclonal antibody against human THP (H-135, no. sc-20631, Santa Cruz Biotechnology). Three antibodies against NRF2 (RnD Systems no. MAB3925, Cell Signaling no. 12721, and proteintech no. 16396-1-AP) were tested but failed to detect NRF2 protein. ECL reagent (GE Healthcare Amersham Biosciences) was used for visualization of bound antibodies. Signal intensities were quantified using ImageQuant (GE Healthcare). Standardization of equal loading was referred to the signal intensities of GAPDH of the corresponding PVDF membrane. Data are shown as means ± SD. Data were analysed by using 1-way ANOVA with Newman-Keuls’s post hoc test. 24-h urine samples, standardized for equal creatinine levels, were used for Western blot analysis to detect urinary UMOD content.

### Immunohistological and ultrastructural analyses

Fixation of kidneys with 4% paraformaldehyde, paraffin embedding and sectioning and histological analyses of kidneys were performed as described previously^4^,^6^. For multicolour immunofluorescence analyses of UMOD with SDHA and UMOD with FIS1, kidneys were fixed in methacarn fixative. Immunohistochemistry was performed using the following primary antibodies: rabbit polyclonal antibody against FIS1 (no. GTX111010, GeneTex), rabbit polyclonal antibody against NRF1 (no. 12381, Cell Signaling), rabbit monoclonal antibody against SDHA (D6J9M, no. 11998, Cell Signaling), and rat monoclonal antibody against mouse uromodulin (clone 774056; R&D Systems). Immunoreactivity was visualized using 3,3-diaminobenzidine tetrahydrochloride dihydrate (DAB) (brown colour). Nuclear counterstaining was done with haemalum (blue colour). Colocalizations of UMOD with NRF1, UMOD with SDHA and UMOD with FIS1 were studied by multicolour immunofluorescence analysis, using the above mentioned primary antibodies. All secondary antibodies were produced in donkey and coupled to FITC or Cy3 (Dianova). Embedding of slides was done with Vectashield antifade solution (Vector Laboratories) containing DAPI as a nuclear counterstain. Fluorescence analyses were performed using a confocal laser scanning microscope (LSM 710, Zeiss).

Fixation of kidneys with 3% glutaraldehyde in PBS (pH 7.2) and procession to ultrathin sections for transmission electron microscopic analyses was performed as described previously^6^.

## Additional Information

**How to cite this article:** Kemter, E. *et al*. Mitochondrial Dysregulation Secondary to Endoplasmic Reticulum Stress in Autosomal Dominant Tubulointerstitial Kidney Disease – *UMOD* (ADTKD-*UMOD*). *Sci. Rep.* **7**, 42970; doi: 10.1038/srep42970 (2017).

**Publisher's note:** Springer Nature remains neutral with regard to jurisdictional claims in published maps and institutional affiliations.

## Supplementary Material

Supplementary Figures

Supplementary Tables

## Figures and Tables

**Table 1:** Selection of functional clusters of differential abundant proteins (DAPs) in ADTKD-*UMOD*, identified by DAVID annotation cluster enrichment analysis.

Enrichment score	Term	counts of DAPs	% of all DAPs
33.29	mitochondrial part	64	30.0
13.59	oxidative phosphorylation	25	11.7
10.85	citrate cycle (TCA cycle)	12	5.6
9.47	endoplasmic reticulum part	17	8.0
6.31	ATP metabolic process	13	6.1
6.31	hydrogen ion transport	9	4.2
6.27	peroxisome	11	5.2
5.67	cation transmembrane transporter activity	11	5.2
4.78	NADH/NADPH oxidoreductase activity	8	3.8
4.47	glucose catabolic process	8	3.8
2.01	extracellular matrix	10	4.7

## References

[b1] Rampoldi L., Scolari F., Amoroso A., Ghiggeri G. & Devuyst O. The rediscovery of uromodulin (Tamm-Horsfall protein): from tubulointerstitial nephropathy to chronic kidney disease. Kidney international 80, 338–347, doi: 10.1038/ki.2011.134 (2011). 21654721

[b2] Eckardt K. U. . Autosomal dominant tubulointerstitial kidney disease: diagnosis, classification, and management–A KDIGO consensus report. Kidney international 88, 676–683, doi: 10.1038/ki.2015.28 (2015). 25738250

[b3] Serafini-Cessi F., Malagolini N. & Cavallone D. Tamm-Horsfall glycoprotein: biology and clinical relevance. Am.J. Kidney Dis. 42, 658–676 (2003). 14520616 10.1016/s0272-6386(03)00829-1

[b4] Kemter E. . No amelioration of uromodulin maturation and trafficking defect by sodium 4-phenylbutyrate *in vivo*: studies in mouse models of uromodulin-associated kidney disease. J Biol Chem 289, 10715–10726, doi: 10.1074/jbc.M113.537035 (2014). 24567330 PMC4036188

[b5] Eckardt K. U. . Autosomal dominant tubulointerstitial kidney disease: diagnosis, classification, and management-A KDIGO consensus report. Kidney international, doi: 10.1038/ki.2015.28 (2015). 25738250

[b6] Kemter E. . Type of uromodulin mutation and allelic status influence onset and severity of uromodulin-associated kidney disease in mice. Hum Mol Genet 22, 4148–4163, doi: 10.1093/hmg/ddt263 (2013). 23748428

[b7] Seo A. Y. . New insights into the role of mitochondria in aging: mitochondrial dynamics and more. Journal of cell science 123, 2533–2542, doi: 10.1242/jcs.070490 (2010). 20940129 PMC2912461

[b8] Kluge M. A., Fetterman J. L. & Vita J. A. Mitochondria and endothelial function. Circulation research 112, 1171–1188, doi: 10.1161/CIRCRESAHA.111.300233 (2013). 23580773 PMC3700369

[b9] Smith J. J. & Aitchison J. D. Peroxisomes take shape. Nat Rev Mol Cell Biol 14, 803–817, doi: 10.1038/nrm3700 (2013). 24263361 PMC4060825

[b10] Mishra P. & Chan D. C. Metabolic regulation of mitochondrial dynamics. The Journal of cell biology 212, 379–387, doi: 10.1083/jcb.201511036 (2016). 26858267 PMC4754720

[b11] Lin J., Handschin C. & Spiegelman B. M. Metabolic control through the PGC-1 family of transcription coactivators. Cell Metab 1, 361–370, doi: 10.1016/j.cmet.2005.05.004 (2005). 16054085

[b12] Zhang Y. & Hayes J. D. The membrane-topogenic vectorial behaviour of Nrf1 controls its post-translational modification and transactivation activity. Sci Rep 3, 2006, doi: 10.1038/srep02006 (2013). 23774320 PMC3684815

[b13] Hardie D. G. AMPK: positive and negative regulation, and its role in whole-body energy homeostasis. Curr Opin Cell Biol 33, 1–7, doi: 10.1016/j.ceb.2014.09.004 (2015). 25259783

[b14] Alexander A. & Walker C. L. The role of LKB1 and AMPK in cellular responses to stress and damage. FEBS letters 585, 952–957, doi: 10.1016/j.febslet.2011.03.010 (2011). 21396365

[b15] Kemter E. . Novel missense mutation of uromodulin in mice causes renal dysfunction with alterations in urea handling, energy, and bone metabolism. Am J Physiol Renal Physiol 297, F1391–1398, doi: 10.1152/ajprenal.00261.2009 (2009). 19692485

[b16] Wang L., Zheng Y., Xu H., Yan X. & Chang X. Investigate pathogenic mechanism of TXNDC5 in rheumatoid arthritis. PLoS One 8, e53301, doi: 10.1371/journal.pone.0053301 (2013). 23326410 PMC3541148

[b17] Gess B. . The cellular oxygen tension regulates expression of the endoplasmic oxidoreductase ERO1-Lalpha. Eur J Biochem 270, 2228–2235 (2003). 12752442 10.1046/j.1432-1033.2003.03590.x

[b18] Bando Y. . ORP150/HSP12A protects renal tubular epithelium from ischemia-induced cell death. Faseb J 18, 1401–1403, doi: 10.1096/fj.03-1161fje (2004). 15240565

[b19] Sullivan D. C. . EndoPDI, a novel protein-disulfide isomerase-like protein that is preferentially expressed in endothelial cells acts as a stress survival factor. J Biol Chem 278, 47079–47088, doi: 10.1074/jbc.M308124200 (2003). 12963716

[b20] Arrington D. D. & Schnellmann R. G. Targeting of the molecular chaperone oxygen-regulated protein 150 (ORP150) to mitochondria and its induction by cellular stress. Am J Physiol Cell Physiol 294, C641–650, doi: 10.1152/ajpcell.00400.2007 (2008). 18094145

[b21] Wu Y. B. . CHOP/ORP150 ratio in endoplasmic reticulum stress: a new mechanism for diabetic peripheral neuropathy. Cell Physiol Biochem 32, 367–379, doi: 10.1159/000354444 (2013). 23988440

[b22] Ventura-Clapier R., Garnier A. & Veksler V. Transcriptional control of mitochondrial biogenesis: the central role of PGC-1alpha. Cardiovascular research 79, 208–217, doi: 10.1093/cvr/cvn098 (2008). 18430751

[b23] Dimitrov L., Lam S. K. & Schekman R. The role of the endoplasmic reticulum in peroxisome biogenesis. Cold Spring Harb Perspect Biol 5, a013243, doi: 10.1101/cshperspect.a013243 (2013). 23637287 PMC3632059

[b24] Quiros P. M., Langer T. & Lopez-Otin C. New roles for mitochondrial proteases in health, ageing and disease. Nat Rev Mol Cell Biol 16, 345–359, doi: 10.1038/nrm3984 (2015). 25970558

[b25] Venkatesh S., Lee J., Singh K., Lee I. & Suzuki C. K. Multitasking in the mitochondrion by the ATP-dependent Lon protease. Biochimica et biophysica acta 1823, 56–66, doi: 10.1016/j.bbamcr.2011.11.003 (2012). 22119779 PMC3263341

[b26] Raturi A. & Simmen T. Where the endoplasmic reticulum and the mitochondrion tie the knot: the mitochondria-associated membrane (MAM). Biochimica et biophysica acta 1833, 213–224, doi: 10.1016/j.bbamcr.2012.04.013 (2013). 22575682

[b27] Gilady S. Y. . Ero1alpha requires oxidizing and normoxic conditions to localize to the mitochondria-associated membrane (MAM). Cell Stress Chaperones 15, 619–629, doi: 10.1007/s12192-010-0174-1 (2010). 20186508 PMC3006622

[b28] Liberti M. V. & Locasale J. W. The Warburg Effect: How Does it Benefit Cancer Cells? Trends Biochem Sci 41, 211–218, doi: 10.1016/j.tibs.2015.12.001 (2016). 26778478 PMC4783224

[b29] Pinti M. . Mitochondrial Lon protease at the crossroads of oxidative stress, ageing and cancer. Cell Mol Life Sci 72, 4807–4824, doi: 10.1007/s00018-015-2039-3 (2015). 26363553 PMC11113732

[b30] Che R., Yuan Y., Huang S. & Zhang A. Mitochondrial dysfunction in the pathophysiology of renal diseases. Am J Physiol Renal Physiol 306, F367–378, doi: 10.1152/ajprenal.00571.2013 (2014). 24305473

[b31] Nunnari J. & Suomalainen A. Mitochondria: in sickness and in health. Cell 148, 1145–1159, doi: 10.1016/j.cell.2012.02.035 (2012). 22424226 PMC5381524

[b32] Rampoldi L. . Allelism of MCKD, FJHN and GCKD caused by impairment of uromodulin export dynamics. Hum. Mol. Genet. 12, 3369–3384 (2003). 14570709 10.1093/hmg/ddg353

[b33] Nasr S. H., Lucia J. P., Galgano S. J., Markowitz G. S. & D’Agati V. D. Uromodulin storage disease. Kidney Int. 73, 971–976 (2008). 18004297 10.1038/sj.ki.5002679

[b34] Huang da W., Sherman B. T. & Lempicki R. A. Systematic and integrative analysis of large gene lists using DAVID bioinformatics resources. Nat Protoc 4, 44–57, doi: 10.1038/nprot.2008.211 (2009). 19131956

