# Supplementary material for: Mitochondrial Dysregulation Secondary to Endoplasmic Reticulum Stress in Autosomal Dominant Tubulointerstitial Kidney Disease – UMOD (ADTKD-UMOD)
Source: Sci Rep. 2017 Feb 21;7:42970. doi: 10.1038/srep42970 (PMC5318959; doi:10.1038/srep42970)
Supplement: Supplementary Figures [file srep42970-s1.pdf]

MITOCHONDRIAL DYSREGULATION SECONDARY TO ENDOPLASMIC RETICULUM STRESS IN AUTOSOMAL DOMINANT TUBULOINTERSTITIAL KIDNEY DISEASE – *UMOD* (ADTKD-*UMOD*)

Elisabeth Kemter, Thomas Fröhlich, Georg J. Arnold, Eckhard Wolf, and Rüdiger Wanke

SUPPLEMENTARY FIGURES

Supplementary Fig. 1

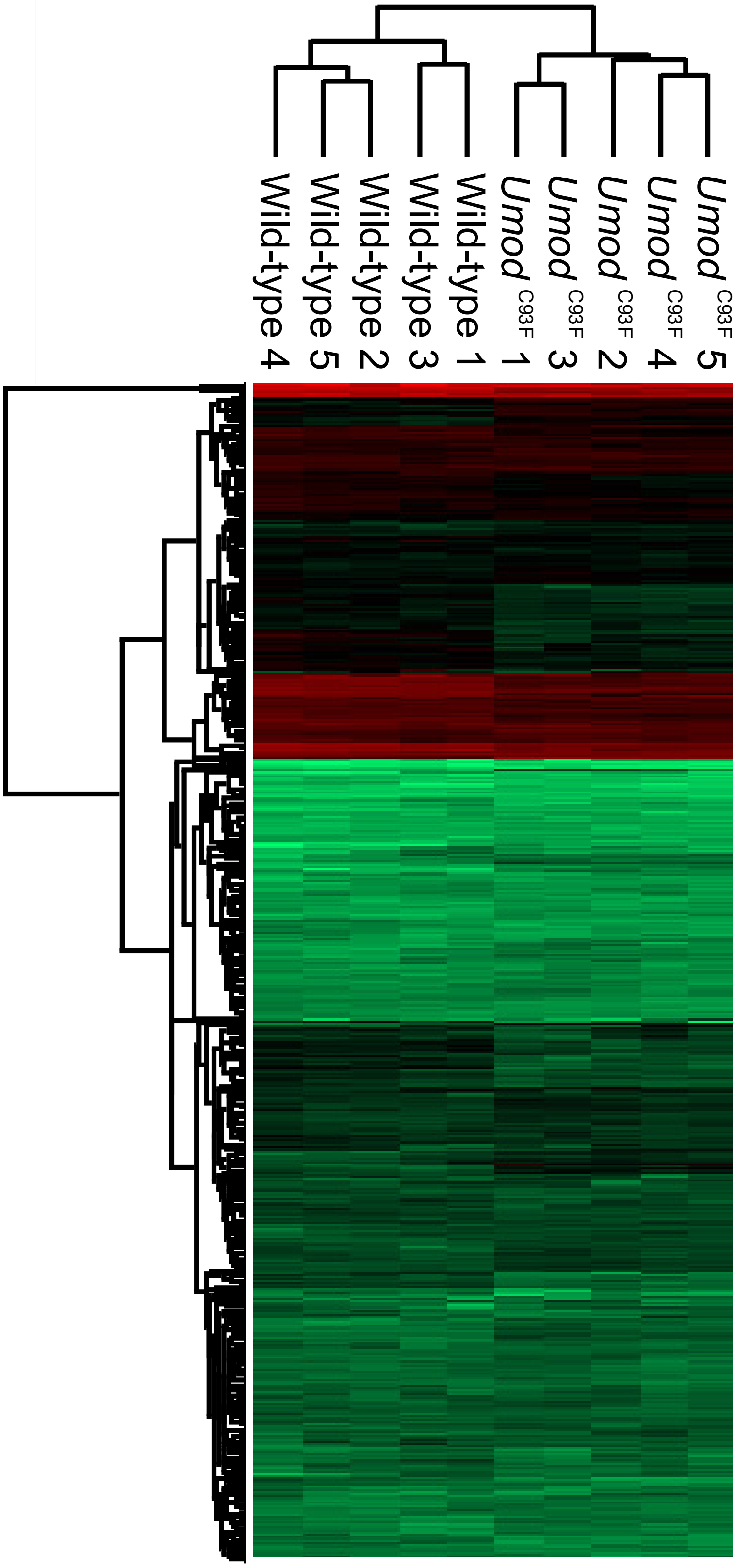

Heat map of LFQ values: Hierarchical clustering of LFQ values clearly separated the two genotype groups .

Supplementary Fig. 2: PROTEIN PROCESSING IN ENDOPLASMIC RETICULUM

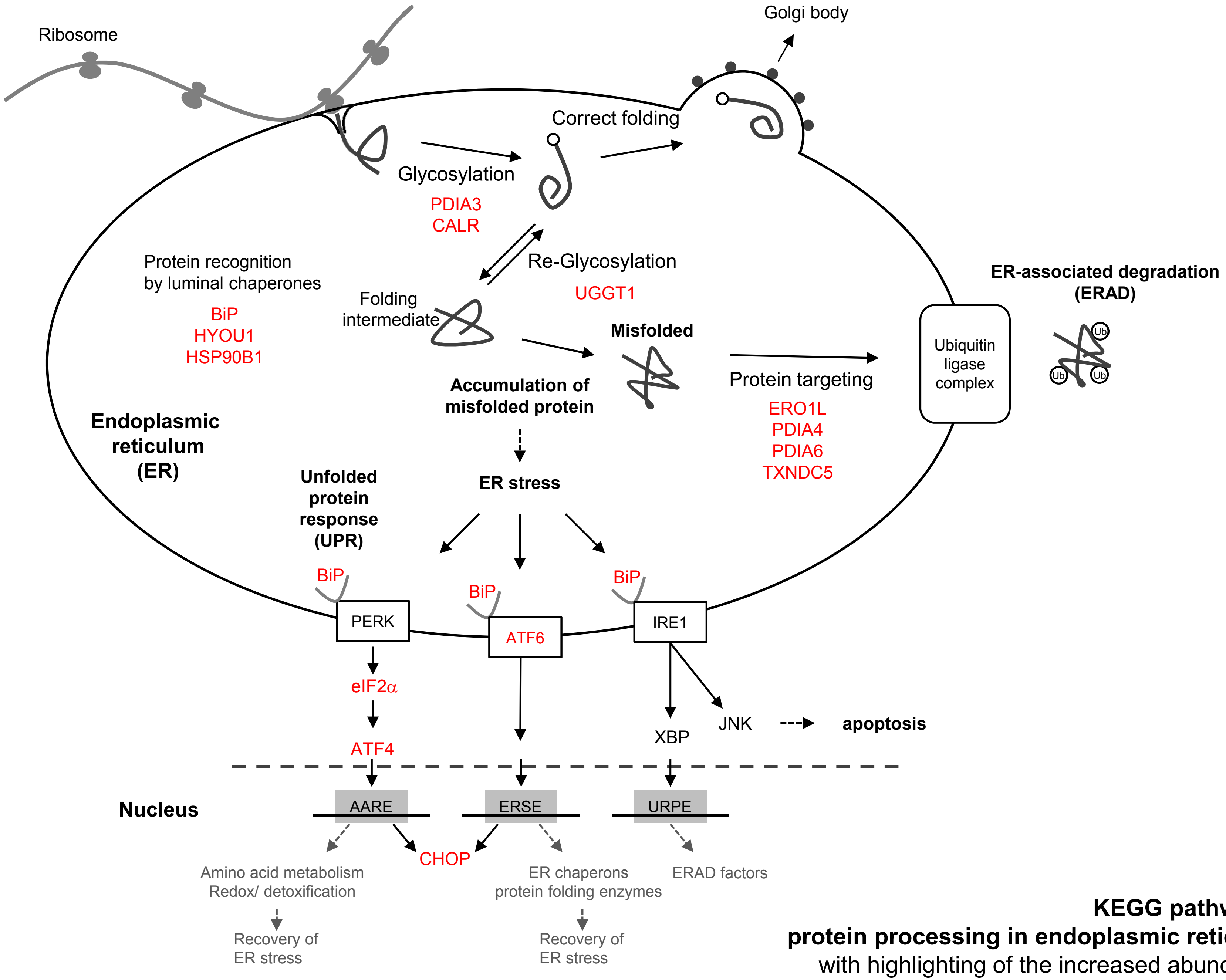

KEGG pathway of
protein processing in endoplasmic reticulum
with highlighting of the increased abundant or activated proteins (in red) in ADTKD-*UMOD.*

Supplementary Fig. 3

A

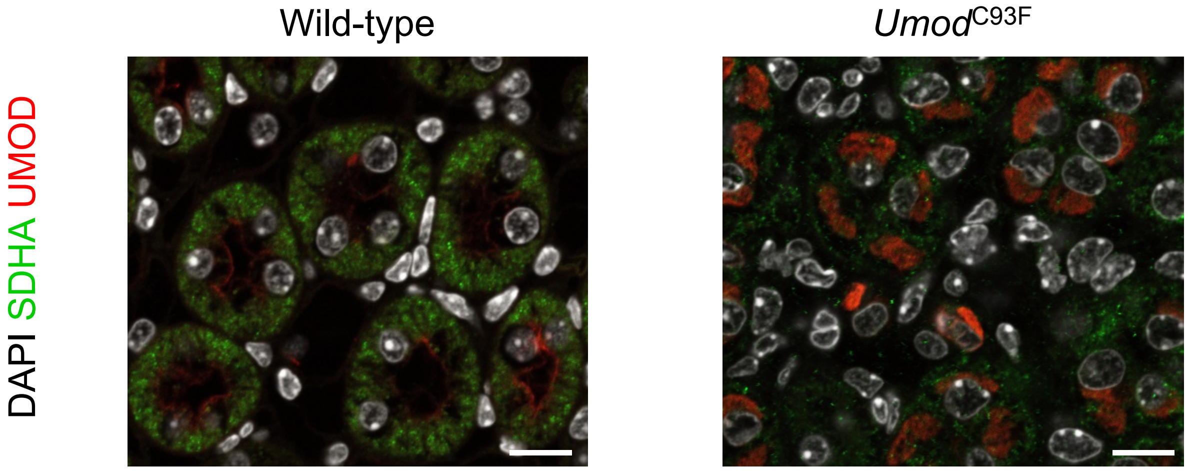

Co-localization of uromodulin and SDHA in TAL cells of a wild-type mouse and a homozygous *Umod*^{C93F} mutant mouse, studied by multicolour immunofluorescence analysis, to demonstrate reduced abundance and altered localization of SDHA in TAL cells in ADTKD-*UMOD*. Age of mice analysed: 4 months. Bar represents 10 μm. DAPI: nuclear marker.

B

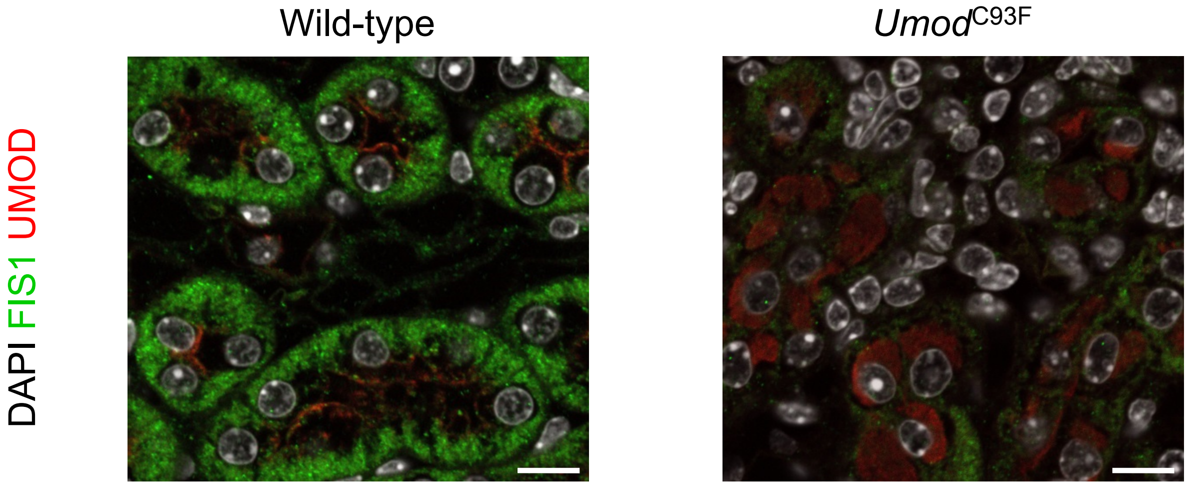

Co-localization of uromodulin and FIS1 in TAL cells of a wild-type mouse and a homozygous *Umod*^{C93F} mutant mouse, studied by multicolour immunofluorescence analysis, to demonstrate reduced abundance and altered localization of FIS1 in TAL cells in ADTKD-*UMOD*. Age of mice analysed: 4 months. Bar represents 10 μm. DAPI: nuclear marker.

Supplementary Fig. 4

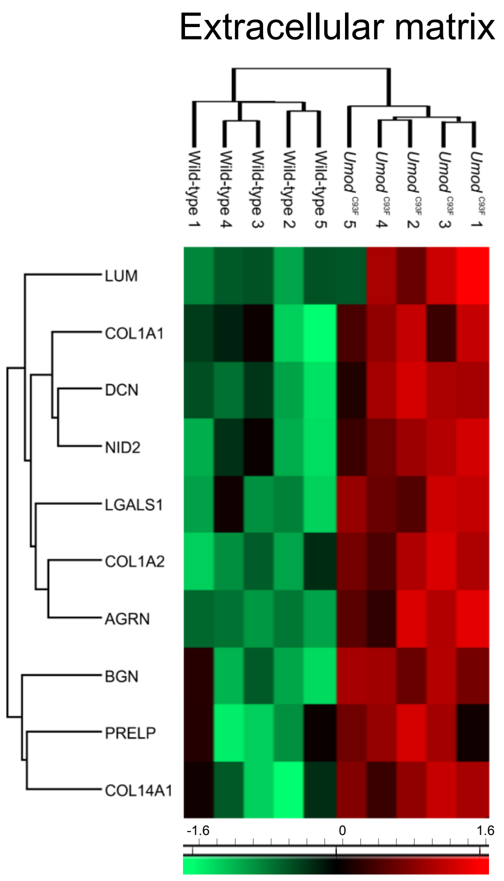

Z score heat map showed differentially abundant extracellular matrix proteins.

Supplementary Fig. 5

A

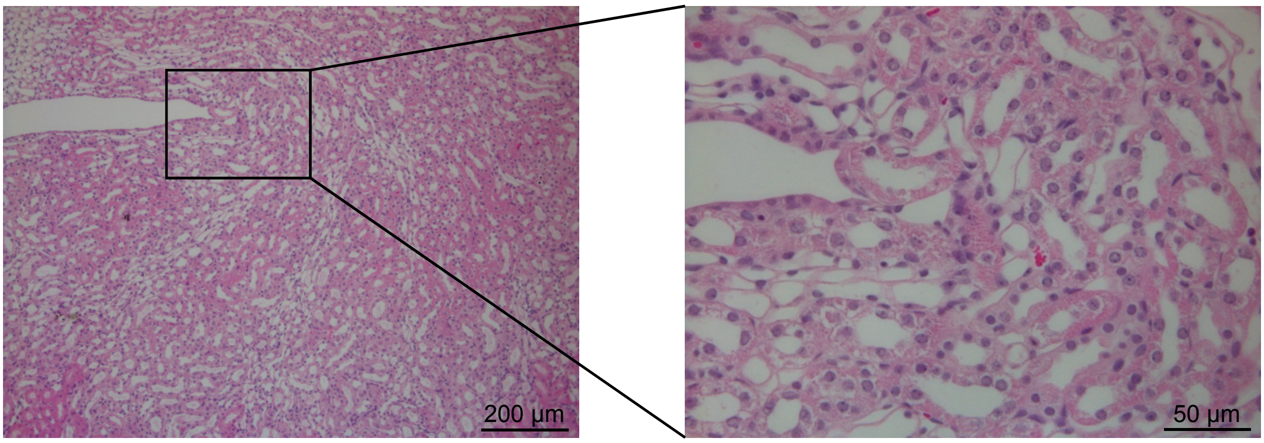

Hematoxylin & Eosin stain of a kidney section of a four-month-old homozygous *Umod*^{C93F} mutant mouse, for demonstration of early disease stage at this age where no progressed morphological kidney alterations like interstitial fibrosis, tubular atrophy, or infiltrates of inflammatory cells are present.

B

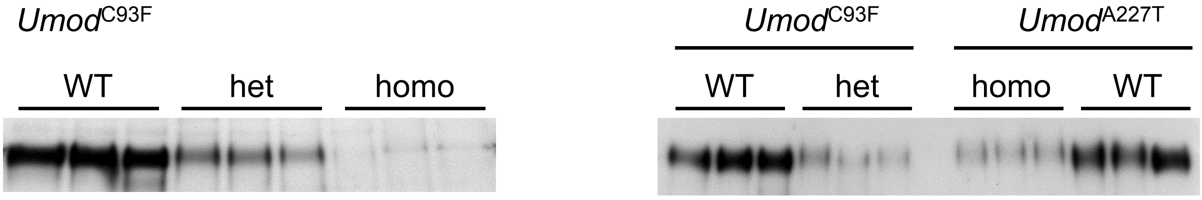

Urine analysis of uromodulin excretion, standardized on equal creatinine content, demonstrates severity of protein maturation defect of uromodulin, which is dependent from kind of mutation and allelic status. Uromodulin excretion of *Umod*^{C93F} heterozygotes and *Umod*^{A227T} homozygous mutants is decreased at a similar range compared to wild-type controls. Uromodulin excretion of *Umod*^{C93F} homozygous mutants is more pronounced decreased compared to *Umod*^{C93F} heterozygous mutants. In conclusion, although *Umod*^{A227T} and *Umod*^{C93F} mutant mice exhibit similar disease phenotype, *Umod*^{C93F} mutant mice are more severely affected by a more pronounced mutant UMOD maturation defect. Homozygous mutant mice exhibit a more pronounced UMOD maturation defect and disease phenotype than heterozygous mutants of the same line.

WT, wild-type; het, heterozygous mutant; homo, homozygous mutant. The corresponding mutant mouse line is indicated.
